# IgG4 Cholangiopathy

**DOI:** 10.1155/2012/472376

**Published:** 2011-08-04

**Authors:** Yoh Zen, Yasuni Nakanuma

**Affiliations:** ^1^Institute of Liver Studies, King's College Hospital, Denmark Hill, London SE5 9RS, UK; ^2^Department of Human Pathology, Kanazawa University Graduate School of Medicine, Kanazawa 920-8640, Japan

## Abstract

IgG4 cholangiopathy can involve any level of the biliary tree which exhibits sclerosing cholangitis or pseudotumorous hilar lesions. Most cases are associated with autoimmune pancreatitis, an important diagnostic clue. Without autoimmune pancreatitis, however, the diagnosis of IgG4-cholangiopathy is challenging. Indeed such cases have been treated surgically. IgG4-cholangiopathy should be diagnosed based on serological examinations including serum IgG4 concentrations, radiological features, and histological evidence of IgG4^+^ plasma cell infiltration. Steroid therapy is very effective even at disease relapse. A Th2-dominant immune response or the activation of regulatory T cells seems to be involved in the underlying immune reaction. It is still unknown why IgG4 levels are specifically elevated in patients with this disease. IgG4 might be secondarily overexpressed by Th2 or regulatory cytokines given the lack of evidence that IgG4 is an autoantibody.

## 1. Introduction

IgG4-related disease is a unique systemic inflammatory condition characterized by tumorous swelling of affected organs and high-serum IgG4 concentrations [1–3]. Autoimmune pancreatitis is a prototype of IgG4 disease as Hamano et al. described in a landmark paper in 2001 [4]. Further studies have confirmed that IgG4-related disease can involve a variety of organs including the salivary glands (chronic sclerosing sialadenitis) [5, 6], lacrimal glands (Mikulicz's disease) [7, 8], and retroperitoneum (retroperitoneal fibrosis) [9, 10]. The bile duct lesion is called IgG4-related sclerosing cholangitis (IgG4-SC) [11] or IgG4-associated cholangitis [12, 13] (the former is used hereafter). Since we reported that IgG4-SC is a distinct entity which should be differentiated from primary sclerosing cholangitis (PSC) [11], clinical and pathological features have been clarified [12, 13]. In this paper, we describe the concept, pathology, differential diagnosis, and pathogenesis of IgG4-SC.

## 2. Spectrum of IgG4 Cholangiopathy

The relationship between IgG4-SC and autoimmune pancreatitis is summarized in Table 1. IgG4-SC can manifest as diffuse sclerosing cholangitis or a hilar pseudotumourous mass [11]. The former should be differentiated from PSC, whereas the latter radiologically resembles hilar cholangiocarcinoma [14]. Of note is that most case of IgG4-SC are associated with autoimmune pancreatitis. A study by the Mayo Clinic found that only 4 of 53 patients (7.5%) with IgG4 cholangiopathy had cholangitis without autoimmune pancreatitis [13]. Whether or not IgG4-SC can involve only peripheral small bile ducts like small-duct PSC is an interesting issue. Given that a recent study revealed that peripheral IgG4 cholangiopathy was always associated with large duct lesions [15], it seems safe to assume that IgG4-SC predominantly affects large bile ducts, which are detectable by cholangiographic or radiological examinations.

Recent papers have introduced IgG4-related autoimmune hepatitis [16], which accounts for 3% of cases of type 1 autoimmune hepatitis in the Japanese population [17]. The term IgG4 hepatitis should be only used for patients who do not have radiological biliary abnormalities and are found to have chronic hepatitis based on liver biopsies. Given that one cases of IgG4 hepatitis was complicated later by sclerosing cholangitis during followup [17], IgG4 hepatitis might also belong to a spectrum of IgG4 cholangiohepatitis. 

## 3. Clinical Features and Autoantibodies

In our experience, patients with IgG4-SC usually present with obstructive jaundice due to a pancreatic head mass (autoimmune pancreatitis) or severe biliary stricture [12, 13]. Other patients are sometimes discovered to have IgG4-SC during a workup for other IgG4-related conditions such as sialadenitis, retroperitoneal fibrosis and kidney lesions. Weight loss or new-onset diabetes mellitus due to pancreatitis is another potential symptom. 

Patients with IgG4 disease share serological abnormalities irrespective of the organ of origin. There is no doubt that elevated serum IgG4 levels are the most specific indicator. Other sensitive but not specific markers include hyper *γ*-globulinemia (observed in 50% of patients), hyper IgG (60–70%), antinuclear antibodies (40–50%), rheumatoid factor (20%), and eosinophilia (15–25%) [18, 19]. Autoantibody against SS-A (Ro) or SS-B (La), antimitochondria antibody, and antineutrophilic cytoplasmic antibody (ANCA) are all exceptional (<5%) [18, 19]. 

Studies on autoimmune pancreatitis have provided further data on autoantibodies which might participate in the pathogenesis. Antibodies against lactoferrin (LF) and carbonic anhydrase (CA) II are frequently detected in cases of autoimmune pancreatitis (73% and 54%, resp.) [20]. Interestingly, a strong positive correlation between increases in serum IgG4 levels and anti-CA-II antibody levels has been reported [21]. Anti-CA-IV, another autoantibody, was detected in 10 of 29 (34%) patients with autoimmune pancreatitis [22]. Given that LF and CAs are expressed in some exocrine organs, these autoantibodies may be related to systemic manifestations of IgG4-related disease. Of note is that autoantibodies of the IgG4 subclass have not been detected in patients with IgG4-related disease so far.

## 4. Diagnosis

### 4.1. Surgical Cases

It is not difficult to make a diagnosis of IgG4-SC if surgically resected specimens are available. The gold standard for the diagnosis of IgG4-SC is histology including characteristic features on H&E and extensive infiltration by IgG4^+^ plasma cells on immunostaining. Pathological features can be summarized as follows: (1) diffuse lymphoplasmacytic infiltration, (2) storiform fibrosis, (3) obliterative phlebitis, (4) eosinophilic infiltration, and (5) numerous IgG4^+^ plasma cells [11, 23]. Features unusual for IgG4-SC are neutrophilic infiltration with or without abscesses, xanthogranulomatous change, and mucosal erosive change. Obliterative phlebitis is a finding characteristic for IgG4-related disease irrespective of the organ affected. We speculate that endothelium may express chemotactic factors, but this has not been examined so far.

### 4.2. Patients with Autoimmune Pancreatitis

Serology, imaging, other organ involvement, and biopsy need to be considered for the diagnosis of nonsurgical cases. Given that most patients (>90%) with IgG4-SC have autoimmune pancreatitis, it seems most important to examine changes in the pancreas. Autoimmune pancreatitis is radiologically suspected in most cases, and the diagnosis can be confirmed by the serological examination of IgG4. Histological detection of IgG4^+^ plasma cells is usually not necessary for cases showing typical radiological features (sausage-like diffuse swelling, peripancreatic capsule-like rim, and irregular narrowing of the pancreatic duct) [24] and high-serum IgG4 levels. But, if there are any unusual features on imaging or IgG4 levels are not elevated, biopsies should be considered to detect IgG4^+^ plasma cells. Most institutions use 135 or 140 mg/dL as a cut-off point for serum IgG4 levels, with more than 300 mg/dL being highly specific for IgG4-related disease [4, 25].

### 4.3. Patients without Autoimmune Pancreatitis

It is challenging to diagnose IgG4-SC not associated with pancreatitis. In fact, most patients have been surgically treated for suspected biliary malignancy [13]. In our experience, detecting IgG4^+^ plasma cell is recommended even if the patients have high-serum IgG4 concentrations. Three potential approaches have been proposed to detect infiltration by IgG4^+^ plasma cells. Vater's ampulla biopsy is least invasive and technically easiest, and especially useful for patients discovered endoscopically to have ampullary swelling [26–29]. Another potential approach is a liver needle biopsy which can detect IgG4^+^ plasma cells infiltrating into peripheral small portal tracts [15, 30, 31]. This is particularly useful for patients with intrahepatic biliary abnormalities on cholangiograms, but not useful for patients with only intrapancreatic bile duct stricture [15]. The last choice is a bile duct biopsy, the usefulness of which might depend on the ability and experience of the endoscopists [29]. The biggest advantage of this method is that not only IgG4^+^ plasma cells but also other histological features such as storiform fibrosis and eosinophilic infiltration are detectable [29]. The specificity and sensitivity of the diagnosis by these three biopsies are summarized in Table 2. Normally, more than 10 IgG4^+^ plasma cells are used as the diagnostic threshold for biopsy samples.

### 4.4. Steroid Trial

There is no international consensus regarding diagnostic steroid trials for IgG4-related disease. Some institutions use steroid trials for the diagnosis [32–34], whereas other groups, especially from Japan, are against doing so [35]. Given that IgG4-SC needs to be differentiated from malignant tumours (cholangiocarcinoma) different from other typical autoimmune diseases, much attention should be paid to diagnostic steroid trials.

## 5. Differential Diagnosis

### 5.1. PSC

**Figure 1:**
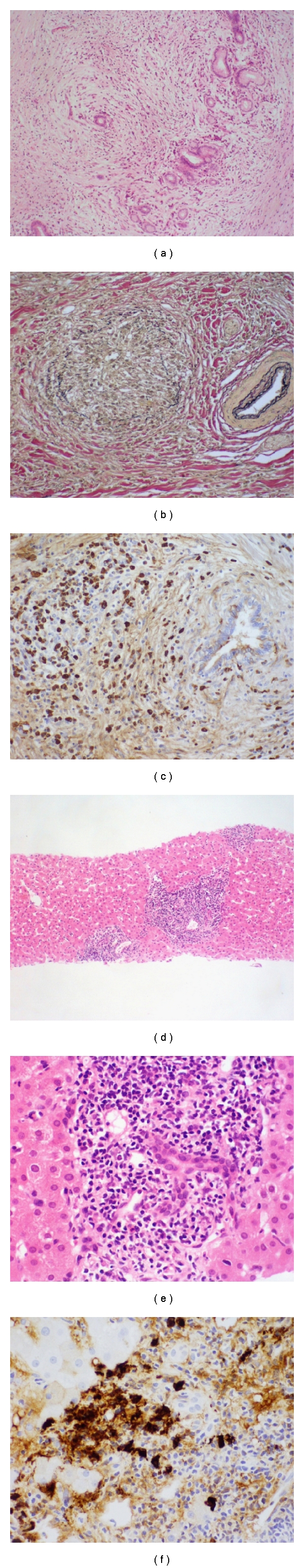
Histopathology of IgG4-related sclerosing cholangitis. The surgical specimens show diffuse inflammatory cell infiltration with fibrosis involving peribiliary glands (a), obliterative phlebitis (b), and infiltration of many IgG4^+^ plasma cells (c). The liver needle biopsy reveals portal inflammation (d), bile duct damage (e), and IgG4^+^ plasma cells (f).

IgG4-SC must be differentiated from PSC given that the steroid responsiveness is completely different. These two entities are histologically distinct. Large bile ducts affected by IgG4-SC show transmural inflammation with storiform fibrosis and obliterative phlebitis (Figure 1), whereas inflammation is more extensive on the luminal side with erosion or xanthogranulomatous changes in PSC [11]. Clinically, a young age (<40 years) and history of inflammatory bowel disease are features suggestive of PSC, whereas IgG4-SC is more likely in patients with other sclerosing lesions including autoimmune pancreatitis and retroperitoneal fibrosis. Serologically, IgG4 levels are most useful, but it should be noted that 9% of PSC patients show elevated IgG4 levels [36]. Positivity for ANCA is suggestive of PSC. Eosinophilia is similarly detectable in both diseases. Nakazawa et al. reported that PSC and IgG4-SC can be differentiated based on a detailed examination of cholangiograms [37]. Liver biopsy is also useful. Infiltration of IgG4^+^ plasma cells or the presence of periportal “fibroinflammatory nodules [30]” in needle biopsy samples is suggestive of IgG4-SC (Figure 1), whereas ductopenia and periductal concentric fibrosis are more commonly seen in PSC [15, 31]. It is still unclear whether or not PSC can be differentiated from “burned-out” IgG4-SC.

### 5.2. PSC with Many IgG4^+^ Plasma Cells

Zhang et al. [38] examined tissue infiltration of IgG4^+^ plasma cells in explanted liver with PSC. Twenty-three of 98 livers (23%) showed more than 10 IgG4^+^ cells per HPF which might be less specific given that most IgG4-related lesions show more than 100 IgG4^+^ cells/HPF in surgical specimens. In addition, another group revealed that 2 out of 41 (5%) explanted livers with PSC showed more than 100 IgG4^+^ cells/HPF [39]. These two studies suggested that explanted livers with PSC can sometimes show moderate degrees of IgG4^+^ cell infiltration (>10 cells/HPF) and rarely exhibit marked infiltration (>100 cells/HPF) around large bile duct lesions. Importantly, the other histological features of these cases were not typical of IgG4-SC but consistent with PSC [38, 39]. The histological diagnosis of IgG4-SC is not enough just based on the number of IgG4^+^ plasma cells.

### 5.3. Follicular Cholangitis

This is a rare disease entity characterized by numerous lymphoid follicles around hilar or perihilar bile ducts [40, 41]. Most patients reported so far underwent surgical resection on suspicion of hilar cholangiocarcinoma. Follicular cholangitis is different from PSC in that the inflammatory cell infiltration is more extensive and biliary epithelial damage is not conspicuous. IgG4^+^ plasma cell infiltration or obliterative phlebitis is usually not conspicuous, different from IgG4-SC [40].

### 5.4. Hilar Cholangiocarcinoma

In our experience, hilar cholangiocarcinoma is the most important and difficult differential diagnosis of IgG4-SC in the clinical field, particularly for patients without autoimmune pancreatitis. Radiological features of IgG4-SC sometimes resemble those of hilar cholangiocarcinoma [14]. Serum IgG4 levels can be mildly elevated in patients with cholangiocarcinoma, but titers of more than 300 mg/dL are highly suggestive of IgG4-SC. As described above, histological examination to detect IgG4^+^ cell infiltration is needed for patients without autoimmune pancreatitis.

## 6. Treatment

IgG4-SC responds dramatically to steroid therapy the same as other IgG4-related lesions. This is one significant difference from PSC [12, 13]. At the moment, it is difficult to conclude the recommended dose and duration of steroid therapy for IgG4-SC because of a lack of published data. A Japanese study on autoimmune pancreatitis recommended an initial dose of 0.6 mg/kg/day, which was then reduced to a maintenance dose over a period of 3–6 months [42]. In that study, disease relapses appeared to be reduced but not eliminated by maintenance treatment with low-dose steroid [42]. Rituximab is a potential treatment for steroid-resistant autoimmune pancreatitis or IgG4-SC [43]. 

## 7. Pathogenesis

Recent papers have provided data suggesting that the Th2-type immune response is activated in IgG4-related disease including IgG4-SC [44–48]. Quantitative real-time PCR using RNA extracted from frozen tissue of affected organs including bile ducts revealed significantly higher ratios of IL-4/IFN-*γ*, IL-5/IFN-*γ*, and IL-13/IFN-*γ* in IgG4-related disease tissues than in tissues from patients with classical autoimmune diseases [49]. Lymphocytes expressing IL-4 were clearly demonstrated by *in situ* hybridization. Recent papers also showed that peripheral blood mononuclear cells collected from patients with IgG4-related disease produced predominantly Th2-type cytokines such as IL-4, IL-5, IL-10, and IL-13 after T-cell stimulation. 

Interestingly, the number of regulatory T-cells (Tregs) is characteristically increased in both tissue and blood of patients with IgG4-related disease. Our investigation revealed that the mRNA expression of forkhead box P3 (Foxp3, a Tregs-specific transcriptional factor) was higher in IgG4-related disease than in classical autoimmune diseases. Two regulatory cytokines, IL-10 and transforming growth factor-*β* (TGF-*β*), are significantly overexpressed [49, 50]. Furthermore, CD4^+^CD25^+^Foxp3^+^ Tregs could be detected within affected tissues by immunohistochemistry, in numbers significantly higher than in autoimmune and nonautoimmune disease controls. The number of Foxp3^+^ cells was significantly correlated with the number of IgG4^+^ plasma cells in IgG4-related cholangitis [51]. Miyoshi et al. examined the number of Tregs in the blood and reported that the number of CD4^+^CD25^high^ Tregs was significantly higher in patients with AIP than in patients with chronic pancreatitis and was correlated with the level of IgG4 in serum [52]. The number of naïve Tregs was significantly decreased. They speculated that hyporeaction of naïve Tregs might be involved in the development of IgG4-related disease, whereas hyperreaction of CD4^+^CD25^high^ Tregs could reflect IgG4-related disease progression [52]. 

The possible involvement of *H pylori* in the pathogenesis of AIP was reported in 2005 [53]. Gastric *H. pylori* infection triggers AIP in genetically predisposed subjects via molecular mimicry between human CA-II and alpha-carbonic anhydrase of *H. pylori *[54]. Frulloni et al. found that 94% of patients with AIP had antibodies against a plasminogen-binding protein of *H. pylori *[55]. The amino acid sequence of the plasminogen-binding protein exhibited homology with that of the ubiquitin-protein ligase E3 component n-recognin 2, an enzyme expressed in pancreatic acinar cells. However, the involvement of *H. pylori* in the pathogenesis of other IgG4-related lesions has not been reported so far.

## 8. Conclusion

IgG4-SC is a unique cholangiopathy which should be differentiated from classical PSC or biliary malignancy. An underlying immune response might be mediated by predominantly Th2 or regulatory cytokines.

## Figures and Tables

**Table 1:** Disease spectrum of IgG4 pancreato-cholangiopathy and differential diagnosis.

IgG4-related pancreato-cholangiopathy	Differential diagnosis
Autoimmune pancreatitis without bile duct involvement	Pancreatic cancer
Autoimmune pancreatitis with IgG4 cholangitis	Pancreatic cancer and cholangiocarcinoma
IgG4-related sclerosing cholangitis	Primary sclerosing cholangitis
IgG4-related sclerosing cholangitis with hilar pseudotumor	Hilar cholangiocarcinoma

**Table 2:** Sensitivity and specificity of detection of IgG4^+^ plasma cells (≥10 cells/high power field) by ampullary, liver, and bile duct biopsies.

	Sensitivity	Specificity	Reference
Ampullary biopsy	80%	100%	[26]
	67%	100%	[27]
	53%	100%	[28]
	52%	91%	[29]

Liver needle biopsy	24%	100%	[30]
	60%	100%	[31]
	26%	100%	[15]

Bile duct biopsy	52%	97%	[29]

## Abbreviations

ANCA: Antineutrophilic cytoplasmic antibody
CA: Carbonic anhydrase
IgG4-SC: IgG4-related sclerosing cholangitis
PSC: Primary sclerosing cholangitis
TGF: Transforming growth factor
Tregs: Regulatory T cells
Foxp3: Forkhead box P3.
